# Demographic Profiles of Adult Trauma During a 5 Year Period (2007-2011) in Kashan, IR Iran

**DOI:** 10.5812/atr.6770

**Published:** 2012-08-21

**Authors:** Mohammad Reza Fazel, Esmaeil Fakharian, Mehrdad Mahdian, Mahdi Mohammadzadeh, Ladan Salehfard, Maryam Ramezani

**Affiliations:** 1 Trauma Research Center, Kashan University of Medical Sciences, Kashan, IR Iran

**Keywords:** Adult, Epidemiology, Wound and Injury

## Abstract

**Background:**

Trauma, in addition to mortality and disability experienced by an individual, imposes direct and indirect economic and social costs on a community. Traditionally, trauma is a disease of young and middle age adults, an age group which is known to be the most dynamic and economically productive of the community. Increasing our knowledge concerning the etiology and patterns of trauma seems to be the most profitable and accessible way to prevent injuries of this nature.

**Objectives:**

This study was designed to evaluate the epidemiology of adult trauma in Kashan, Iran.

**Patients and Methods:**

The current study used a retrospective cross-sectional approach, enrolling all trauma adults (20 - 60 y) admitted to the Shahid Beheshti Hospital, Kashan, between 2007 and 2011. Age, gender, place of residence, work status, educational level, urban/rural location of the accident, method of transportation to hospital, injured body areas of the victims and therapeutic interventions, were extracted from the data registry and analyzed through descriptive statistics using SPSS software.

**Results:**

A total of 22 564 patients were included in this study. Mean age of the victims was 33.18 ± 10.90 years and the male/female ratio was 4:1. Most of the victims were manual workers (61%), and they had completed primary and junior high school level education (49.4%), they were also more likely to be residents of urban areas (88.6%). Regarding the place of injury, most accidents occurred on city streets (43.8%). Approximately 40% of the total victims were transferred to the hospital by emergency medical services (EMS). During the study period, 260 deaths were recorded and among these, 76% were related to traffic accidents.

**Conclusions:**

Regarding the high prevalence of trauma found in manual workers with low educational levels and motorbike users, the establishment of an integrated program aimed at improving public knowledge on the use of safety and protective measures in work environments should be implemented. The use of safety protective equipment by cyclists, motorbikers and car passengers should also be enforced.

## 1. Background

Trauma is the sixth leading cause of death and the fifth leading cause of moderate and severe disability worldwide, it accounts for 10% of all mortalities (1) thus constituting a serious public health problem with significant social and economic costs (2). According to official statistics, road traffic crashes kill nearly 28,000 people in Iran, and injure or disable 300,000 or more each year (3). In recent years, there has been a steady increase in the number of road traffic injuries, and data for the 6 year period (1995–2000) indicates that there has been an estimated 8% annual increase in mortality due to road traffic injuries (4). On the other hand in many developing countries including Iran, documentation of health statistics is poor and information is limited, and as a result the effects of trauma on the population are poorly understood (5). Knowing more about the characteristics of these types of injuries may lead to the development of better strategies in the prevention and improvement of care for trauma patients.

## 2. Objectives

This study was designed to describe the characteristics of injury in adult trauma patients (20-60 y) who were admitted to the Shahid Beheshti Hospital during a five year period. It is hoped that this study will provide an insight into the epidemiology of adult trauma in one of the central Iranian cities, with a population of approximately 400,000.

## 3. Patients and Methods

The present study has been approved by the Research Deputy of the Kashan University of Medical Sciences and the Trauma Research Center Committee. The design was retrospective and the study population comprised all trauma patients aged 20-60 year who were admitted to the Emergency Department of the Kashan Shahid Beheshti Hospital during a period of 5 years (2007-2011). After the establishment of the Trauma Research Center in this university (2003), all information about trauma patients and casualties was entered into a computer using a database from SPSS, Version 16. To perform the present study, the information extracted from the database were; age, sex, work and educational level of the patients, injured body area, mechanism, type and Place of accident, transportation to hospital, patients’ residency (urban or rural), type of treatment performed on the patients (outpatient or inpatient) and the cost of treatment.

## 4. Results

A total of 22 564 trauma patients were included in the study. Demographic data are presented in Table 1. Mean age was 33.18 ± 10.90 years, and the male/female ratio was 4:1. Hospitalization was indicated in 21 118 cases (93.6%). The length of hospitalization was from one to 83 days (average 3.5 days) and the average hospitalization cost per day was approximately $116. Regarding the patients’ place of residence, 88.6% lived in urban areas and the remaining (11.4%) were rural residents. The majority of the patients (60%) were conveyed to the hospital by non-specialized vehicles (i.e. private vehicles) while, approximately 40% were transferred by EMS. The most common type of injury was traffic accidents accounting for 60.4% of the patients, followed by injuries in the home and workplace (Table 1). The most commonly affected body regions injured were the extremities (58.8%), followed by the head, neck and spine (31.2%) (Table 2). The educational level of the patients was also studied. The majority of patients (49.4%) were educated to primary and junior high school levels and a small minority (4.1%) was illiterate (Table 3). The number of deaths recorded during the study period was 260, and 76% of these were related to traffic accidents.

**Table 1.** Demographic Data of The Trauma Victims

	Patient, %
**Occupation**	
Manual worker	61
Sedentary work	4.5
Farmer	1.5
Student	4.8
Housekeeper	16.8
Unemployed	1.5
Other	9.9
**Place of Injury**	
Workplace	15.1
Home	19.7
Street	43.8
Local road	12.1
Outer-city highway	4.5
Sports place	1.4
Other places	3.4

**Table 2.** Injured Body Areas of Trauma Victims

	Portion, %
Ear, nose, throat	7.2
Head, neck, spine	31.2
Chest	0.9
Abdomen	1.4
Pelvis and urinary tract	0.3
External genital organs	0.2
Extremities	58.8

**Table 3.** Educational Level of Trauma Victims

		Amount, %
Illiterate	4.1
Primary and junior high school	49.4
Senior high school	39.6
Associate degree	6.2
Master's degree or higher	0.2
Unknown	0.5
Total	100

## 5. Discussion

Trauma is a major cause of mortality and morbidity in young and middle age adults (20 – 60 y). This age group is considered to be the most economically productive and constitutes the majority of Kashan’s households. The adult population of Kashan accounts for 77% (n = 314, 394) of the region’s total population (n = 400,000). In this present study, the average age at the time of injury was 33.18 ± 10.9 which is similar to results from other studies conducted in Iran (4) and the world (6-8). The male/female ratio in the present study was 4:1 and this is also comparable to other related studies (4, 5). The percentage of Kashan’s adult population who were hospitalized in Shahid Beheshti Hospital during 2009 was 1.5% and the rate of injury in the adults was 1 245 per 100,000 population per year, in contrast, the worldwide rate of unintentional injuries is 61 per 100,000 (9) indicating a much higher number of injuries than the global rate. Overall, more than half of the trauma victims, were manual workers and due to the fact that Kashan is a semi-industrial city, this finding is understandable. In addition, half of the traffic accidents and nearly 70% of the occupational accident victims were manual workers. During the study period, 260 deaths were recorded among the trauma victims, 76% were related to traffic accidents and 74% of the death toll consisted of motorcycle drivers. Since low income workers in this city are the most frequent users of motorcycles, these results are not unexpected. Educational levels of 50% of the victims were to the levels of primary and junior high school, and this is also consistent with the findings of Zargar et al. (5). The number of victims who were urban residents was eight times the number of rural fatalities. Due to increased migration from the rural areas to urban zones and a decline in the rural population, this result seems reasonable. In our study, road traffic accidents were the most common cause of injury, especially among hospitalized patients. Most of these accidents occurred on city streets and by motorcyclists. Our findings are contrary to Caixeta’s report, in his study the majority of victims were pedestrians and car passengers (10). As mentioned earlier, Kashan is a semi-industrialized small city and the use of motorcycles is very popular, this may explain the difference with Caixeta’s results.

According to this five year study, 40% of the victims were transported to the hospital by EMS. Patient transportation by ambulance increased from 11% in 2004 to 22% in 2009 in Kashan City. Coverage of ambulance transportation in Iran is 7.5% overall (11). Therefore, patient transportation by ambulance in Kashan is higher than the average in Iran, nonetheless, compared to advanced western countries it is lower. However, 14% of patients who had accidents at home and 12% of the occupational accident victims were transported by ambulance. Therefore, educating the general public about the transportation of home and occupational trauma victims appears to be necessary. Extremities injuries were the most common type of injury in this study. Several studies have reported the frequency of limb injury among trauma victims (12-14) and these are consistent with our results. The second most frequent trauma category in this study consisted of the head, neck and spinal injury cases. For motorcyclists, a collision with a motor vehicle is the major cause of traumatic brain injury (TBI) and death. About half of the victims who died due to a TBI in this study, were motorcyclists who had sustained head trauma. On one hand, Kashan’s city streets are generally narrow and have a high traffic volume, but on the other hand, the high number of motorcycle riders not using safety equipments (e.g. helmets), may be the explanation behind the high mortality and morbidity levels. As previously mentioned, most cyclists in Kashan are manual workers with low education levels and low incomes, encouraging the use of helmets along with legislation, combined with education and the free distribution of helmets would be advantageous. The average length of hospital stay in our study was 3.5 days which is less than many other reports (15, 16). This difference may be due to the inclusion of only young and middle age adults in this study, rather than all age groups, which have been studied previously (15). The average cost of hospitalization per day in this study was $116, and adult trauma in 2009 was estimated to cost a total of 4.5 million dollars. According to a UNICEF report, traffic fatalities cost Iran’s economy six billion US dollars every year, which amounts to more than 5% of the country’s Gross National Product (3). As indicated by our findings and also the sheer number of patients needing trauma care services, it seems that an integrated program should be established in order to improve public knowledge of the use of safety and protective measures in work environments. It also highlights the importance of using protective equipment for cyclists, motorbikers and car passengers, especially helmets for motorbikers and cyclists. Finally, legislation of helmet usage, and improvements to the patient transport system in conjunction with education of the population in the use of this service would be of great value. 
